# The Independent Associations between Walk Score^®^ and Neighborhood Socioeconomic Status, Waist Circumference, Waist-To-Hip Ratio and Body Mass Index Among Urban Adults

**DOI:** 10.3390/ijerph15061226

**Published:** 2018-06-11

**Authors:** Gavin R. McCormack, Anita Blackstaffe, Alberto Nettel-Aguirre, Ilona Csizmadi, Beverly Sandalack, Francisco Alaniz Uribe, Afrah Rayes, Christine Friedenreich, Melissa L. Potestio

**Affiliations:** 1 Department of Community Health Sciences, Cumming School of Medicine, University of Calgary, Calgary, AB T2N 4Z6, Canada; ambortni@ucalgary.ca (A.B.); alberto.nettel-aguirre@albertahealthservices.ca (A.N.-A.); icsizmad@ucalgary.ca (I.C.); mlpotest@ucalgary.ca (M.L.P.); 2 Department of Pediatrics, Cumming School of Medicine, University of Calgary, Calgary, AB T3B 6A8, Canada; 3 Department of Oncology, Cumming School of Medicine, University of Calgary, Calgary, AB T2N 4N1, Canada; 4 Faculty of Environmental Design, University of Calgary, Calgary, AB T2N 1N4, Canada; sandalack@ucalgary.ca (B.S.); falanizu@ucalgary.ca (F.A.U.); 5 Urban Design and Heritage, City of Calgary, Calgary, AB T2P 2M5, Canada; Afrah.Rayes@calgary.ca; 6 Department of Cancer Epidemiology and Prevention Research, CancerControl Alberta, Alberta Health Services, Calgary, AB T2S 3C3, Canada; christine.friedenreich@albertahealthservices.ca

**Keywords:** walkability, weight, obesogenic, neighborhood, obesity

## Abstract

*Background*: Environmental and policy factors can influence weight status via facilitating or discouraging physical activity and healthy diet. Despite mixed evidence, some findings suggest that the neighborhood built environment, including “walkability”, is associated with overweight and obesity. Most of these findings have measured body mass index (BMI), yet other weight status measures including waist circumference (WC) and waist-to-hip (W-H) ratio are also predictive of health outcomes, independent of BMI. Our study aim was to estimate the associations between walkability, measured using Walk Score^®^, and each of WC, W-H ratio, and BMI among urban Canadian adults. *Methods*: In 2014, *n* = 851 adults recruited from 12 structurally and socioeconomic diverse neighborhoods (Calgary, Alberta, Canada) provided complete data on a physical activity, health and demographic questionnaire and self-reported anthropometric measures (i.e., height and weight, WC and hip circumference). Anthropometric data were used to estimate WC, W-H ratio, and BMI which were categorized into low and high risk in relation to their potential adverse effect on health. WC and BMI were also combined to provide a proxy measure of both overall and abdominal adiposity. Multivariable logistic regression models estimated odds ratios (OR) and 95% confidence intervals (CI) for associations between each weight status outcome and Walk Score^®^. *Results*: A one-unit increase in Walk Score^®^ was associated with lower odds of being high-risk based on WC (OR = 0.99; 95%CI 0.97–0.99). Notably, those residing in socioeconomically disadvantage neighborhoods had significantly higher odds of being high risk based on WC, BMI, and WC-BMI combined compared with advantaged neighborhoods. *Conclusions*: Interventions that promote healthy weight through the design of neighborhoods that support and enhance the effect of physical activity and diet-related interventions could have a significant population health impact.

## 1. Introduction

Obesity is a risk factor for type 2 diabetes, hypertension, coronary heart disease, some cancers, and musculoskeletal and psychological conditions [1,2,3,4]. In addition, excess weight can constrain functional activities of daily living impacting on quality of life and further promoting weight gain [5]. In 2008, approximately 9.8% of men and 13.8% of women globally were obese, representing a two-fold increase in prevalence since 1980 [6]. The health care costs associated with treating and managing excess weight and obesity-related chronic diseases and the costs associated with the loss of workplace productivity resulting from weight-related absenteeism, disability, presenteeism, and premature mortality place a significant economic burden on society [7,8,9]. Notably, the combined increase in waist circumference (WC), a proxy measure of abdominal fat and BMI is positively associated with increased health care consumption and health care costs [10,11].

The determinants of overweight and obesity are multifaceted [12] and include complex interactions between biological, behavioral, physical environmental, economic, policy, and socio-cultural factors that are often exemplified in socioecological conceptual frameworks [13]. Physical environmental and policy factors in particular can influence overweight and obesity via enabling or discouraging physical activity and healthy diet behavior [14]. Changes in the neighborhood built environment may have contributed obesity epidemic by facilitating the overconsumption of energy-dense foods and inhibiting energy expenditure through discouraging opportunities for physical activity and healthy diets [15]. Creating health supportive environments, including designing and building “leptogenic” or weight reducing neighborhoods, is a strategy that could enable healthy behaviors and promote healthy weight and result in small shifts in overweight and obesity prevalence at the population level [14].

Neighborhood built environments that have high levels of walkability (i.e., a mix of land uses and destinations, connected pedestrian networks, high residential density, and pedestrian-friendly streets and infrastructure) are associated with higher levels of physical activity [16,17,18]. Not surprisingly, given the existing relations between the built environment and physical activity [16,17,18] and diet [19,20], neighborhood environments should also be associated with weight status. However, there is mixed evidence regarding the associations between neighborhood built environment and weight status in adults [21,22,23,24]. Mayne et al. [24] in a review of natural experiments found little evidence regarding number of studies or studies with significant associations between environmental changes and weight outcomes. Nevertheless, neighborhood walkability and proximity and presence of local shops and services are found to be the most consistently associated with weight status [22]. For instance, Walk Score^®^, a publicly available standardized measure of walkability that applies the same algorithm to estimate walkability objectively regardless of geographical location, is found to be associated with weight status [25,26,27,28]. Although Sriram et al. [25] found no association between BMI and Walk Score^®^ after covariate-adjustment, they did observe lower WC among postmenopausal women residing in high versus low walkable neighborhoods. Hirsch et al. [27] found that older adults who relocated to a neighborhood with a higher Walk Score^®^ decreased their BMI and Chui et al. [28] found a lower odds of BMI-assessed overweight and obesity associated with residing in neighborhoods with higher Walk Score^®^ among a sample of Canadian adults.

Other characteristics of the neighborhood environment, such as socioeconomic status might also be associated with weight status [29,30,31,32]. Health behaviors that adversely influence weight status, including low physical activity levels and poor diet are found to be associated with living in lower socioeconomic status areas [33,34,35,36,37]. Socioeconomically advantaged neighborhoods are often more supportive of or provide more opportunities for physical activity and healthy diet while the opposite is often observed for disadvantaged neighborhoods [14,38,39,40]. Thus, to estimate the independent association of neighborhood walkability on weight status, it is important to statistically control for neighborhood socioeconomic status in addition to individual-level sociodemographic characteristics.

Measures of weight status in studies investigating the associations between the neighborhood built environment and obesity have, for the most part included BMI, typically estimated from self-reported height and weight [21,22,23,24]. BMI, however, does not provide direct information about adiposity nor the distribution of adipose tissue within the body. While BMI has been associated with chronic disease and mortality, the health risks of obesity are thought to be underestimated when visceral adiposity is not accounted for in the analysis [41,42,43]. Notably, Lee et al. [44] in their longitudinal study (approximately 6-year follow-up) found higher intersection and food store density, and lower greenspace to be associated with smaller increases in abdominal visceral adiposity. Moreover, in cross-sectional analysis from the same study, more greenspace was associated with lower BMI and WC [44]. WC and hip circumference individually and combined are proxy measures for abdominal or visceral adiposity. Supplementing BMI with other measures of weight status, such as WC and waist-to-hip (W-H) ratio, is important to improve understanding of how neighborhood walkability is associated with weight status and subsequently obesity-related chronic disease. Therefore, the aim of our study was to estimate the associations between neighborhood walkability (Walk Score^®^), independent of neighborhood socioeconomic status and individual-level characteristics, and, WC, W-H ratio, and BMI among urban Canadian adults. 

## 2. Methods

### 2.1. Study and Sample Design

The study method has been previously described [45]. Briefly, using stratified random sampling we selected 12 of 195 established Calgary neighborhoods (built prior to the early 1980s) from strata defined by block pattern (grid, warped grid, and curvilinear) and quartile of socioeconomic status (SES). In Calgary, neighborhoods with grid block patterns are more walkable than warped-grid and curvilinear neighborhood designs [46,47]. Estimation of neighborhood socioeconomic status was informed by previous Canadian research [48], using Statistics Canada Census dissemination area level data (i.e., proportion of those 25–64 years of age, less than high school diploma; proportion of single-parent families; proportion of people renting private dwellings; proportion of divorced, separated, or widowed among those ≥15 years of age; proportion unemployed among those ≥25 years of age; median gross household income; and average value of dwellings). Values for each variable were converted to z-scores and these scores aggregated to create a SES index for each Calgary neighborhood. Neighborhoods were grouped into quartiles based on their SES index for the sampling frame. Calgary (Alberta, Canada) is a city with a population of approximately 1.5 million (in 2018) residing in a geographical area of over 800 km^2^, Calgary is located east of the Rocky Mountains and has an elevation of over one-kilometer above sea level.

Within the 12 neighborhoods, a random sample of 10,500 households was mailed a survey package in April 2014. One adult (≥20 years of age) per household, with the next birthday, was invited to complete two self-administered online questionnaires including: (1) a physical activity, health and demographic questionnaire (PAHDQ); and (2) the Canadian Diet History Questionnaire II (C-DHQ II). Participants were also asked to report their height and weight and measure their WC and hip circumference (HC) using a body tape measure included in the survey package. Participants completed online (*n* = 918) or paper versions (*n* = 105) of the PAHDQ, resulting in a 10.1% response rate (excluding *n* = 407 non-deliverable survey packages). Of the *n* = 1023 respondent, the current study includes *n* = 851 participants who provided complete PAHDQ and body circumference data. C-DHQII results have been presented elsewhere [45]. This study complies with the Helsinki Declaration and was granted ethics approval by The University of Calgary Conjoint Health Research Ethics Board. Informed consent was obtained from all study participants (# REB13-0301).

### 2.2. Variables

#### 2.2.1. Weight Status

Participants were provided with written instructions and diagrams explaining how to measure their waist and hip circumference using the clinical grade measuring tape (Medline, Model NON171333) provided in their study package. Participants were instructed to measure their WC with the tape placed 2 cm above their navel and to measure their HC at the largest location between their hips and thighs. Participants measured and recorded their WC and HC twice (a third measure was requested if the difference between the first two measurements was >0.50 cm). Average WC and HC were estimated and used to calculate W-H ratio. High levels of consistency have been found between self-measured and technician-measured WC and HC [49,50,51]. Participant reported height and weight was used to estimate BMI. All weight status measures were categorized based on established risk cut-offs [52]. Men with a WC ≥102 cm and women with a WC ≥88 cm were classified as high risk. Men with a W-H ratio ≥0.90 and women with a W-H ratio ≥0.85 were classified as high risk. BMI was classified into healthy weight (<25 kg/m^2^); overweight (25 to 29 kg/m^2^), and; obese (≥30 kg/m^2^).

As done in previous studies [53,54,55], we combined WC and BMI to create an additional weight-related risk outcome. Participants were initially classified into five categories: (1) *low risk*: healthy BMI (<25 kg/m^2^) and WC <102 cm (men) or <88 cm (women); (2) *increased risk*: healthy BMI (<25 kg/m^2^) and WC ≥102 cm (men) or ≥88 cm (women) OR overweight BMI (25.0–29.9 kg/m^2^) and WC <102 cm (men) or <88 cm (women); (3) *high risk*: overweight BMI (25–29.9 kg/m^2^) and WC ≥102 cm (men) or ≥88 cm (women) or obese BMI (30.0–34.9 kg/m^2^) and WC <102 cm (men) or <88 cm (women); (4) *very high risk*: obese BMI (30.0–34.9 kg/m^2^) and WC ≥102 cm (men) or ≥88 cm (women); and (5) *extremely high risk*: severely obese BMI (≥35 kg/m^2^).

#### 2.2.2. Neighborhood Walkability

Participant household postal codes were assigned a Walk Score^®^. Walk Score^®^ is an objective indicator of walkability measured on a scale from 1 to 100, with increasing scores reflecting higher walkability. Walk Score^®^ represents the estimated distance from households to nine commonly used amenities (grocery stores, restaurants, shopping, coffee shops, banks, parks, schools, bookstores, and entertainment). Using a decay function, the walkability scores are weighted based on the distance to each amenity and summed. Walk Score^®^ is positively associated with other neighborhood walkability indices [56,57].

#### 2.2.3. Sociodemographic and Health Covariates

Sociodemographic covariates captured from the PAHDQ included participant’s age, sex, ethnicity (white or other), highest education attained (high school or less, college, or university), gross annual household income (<$60,000, $60,000 to 119,000, ≥$120,000, or don’t know/refused to answer), marital status (married/common law or other), number of children at home <18 years of age (at least one or none), dog ownership in past 12 months (owner or non-owner), and motor vehicle access (always/sometimes or never/don’t drive). Health covariates included smoking cigarettes or tobacco in the past 12 months (daily/occasionally or not at all), typical daily amount time spent sleeping, self-reported mental health and self-reported physical health (poor, fair, good, very good, excellent), and attempts to modify weight in the past 12 months via diet, physical activity, supplements, or surgery (attempted versus no attempt).

### 2.3. Statistical Analysis

Descriptive analyses (means and standard deviations (SD) for continuous/numerical variables and frequencies for categorical variables) were undertaken for all participants with complete data. For the inferential analysis, all weight outcomes were dichotomized—WC high vs. low risk, W-H ratio high vs. low risk, WC-BMI high vs. low risk (combination of increased to extreme high risk groups), and BMI overweight (including obese) vs. healthy weight. Multivariable logistic regression models were then used to estimate odds ratios (OR) with corresponding 95 percent confidence intervals (95% CI) for the associations between Walk Score^®^, neighborhood SES, and each of the six weight status outcomes. Interactions between Walk Score^®^ and neighborhood SES were also tested however, none were statistically significant and subsequently not presented here. For each weight status outcome, we estimated two models: (1) a Walk Score^®^ and neighborhood SES adjusted only model; and (2) a Walk Score^®^, neighborhood SES, and sociodemographic and health covariate adjusted model. Pseudo R-square was estimated for all models. Analyses were conducted using IBM Statistical Package for Social Sciences (SPSS; Version 21, IBM Corporation, Armonk, NY, USA).

## 3. Results

### 3.1. Sample Characteristics

The mean age of participants was 53 (SD 14.3) years (Table 1). The sample included high proportions of women (62.4%), Caucasians (73.2%), non-smokers (84.7%), and those with a university education (73.2%), annual household income ≥$120,000 (44.1%), married or common-law (77.3%), no children at home (69.0%), non-dog owners (67.3%), having access to a motor vehicle always or sometimes (86.6%), and attempting to modify their weight in the past year (51.0%).

Approximately one-quarter of participants were classified as high risk based on their WC (23.9%) and one-half (52.2%), were classified as high risk based on their W-H ratio (Table 1). Among our sample, 59.5% were healthy weight, 30.2% were overweight, and 10.3% were obese. Based on the composite WC and BMI variables 56.6% were considered at least risk, 19.6% at an increased risk, 14.5% at high risk, 4.7% at very high risk, and 4.6% at extremely high risk. The mean Walk Score^®^ was 60.3 (SD 15.0) and Walk Score^®^ was higher among participants residing in socioeconomic disadvantaged compared with socioeconomic advantaged neighborhoods (63.0 SD 14.4 vs. 56.9 SD 14.9, *p* < 0.05).

### 3.2. Correlates of waist circumference

Higher Walk Score^®^ was associated with lower odds of having a high-risk WC (OR = 0.98; 95% CI 0.97–0.99) while residing in socioeconomic disadvantaged neighborhoods was associated with higher odds of having a high-risk WC (OR = 1.85; 95% CI 1.32–2.59) (Table 2). After adjusting for all sociodemographic and health covariates, both Walk Score^®^ and neighborhood socioeconomic deprivation remained significantly associated with WC (fully-adjusted model pseudo R^2^ = 0.28). In addition, WC was associated with age (age: OR = 1.25; 95% CI 1.13–1.37 + age^2^: OR= 0.99; 95% CI 0.99–0.99), self-reported physical health (OR = 0.45; 95% CI 0.36–0.57), and attempts to modify weight in the past year (no attempt: OR = 0.37; 95% CI 0.26–0.54).

### 3.3. Correlates of Waist-to-Hip Ratio

Higher Walk Score^®^ was associated with lower odds of having a high-risk W-H ratio (OR = 0.99; 95% CI 0.98–0.99) adjusting for neighborhood socioeconomic deprivation (Table 2). However, Walk Score^®^ was no longer significantly associated with W-H ratio after adjustment for all sociodemographic and health covariates. Adjusting for all other covariates, sex (men: OR = 7.92; 95% CI 5.50–11.41), hours of daily sleep (OR = 0.81; 95% CI 0.69–0.96), better self-reported physical health (OR = 0.74; 95% CI 0.60–0.92), and attempts to modify weight in the past year (no attempt: OR = 0.61; 95% CI 0.44–0.84) were associated with W-H ratio (fully-adjusted model pseudo R^2^ = 0.38).

### 3.4. Correlates of BMI

Higher Walk Score^®^ was associated with lower odds of being overweight or obese (OR = 0.98; 95% CI 0.97–0.99), while residing in a socioeconomic disadvantaged neighborhood was associated with increased odds of being overweight or obese (OR = 1.58; 95% CI 1.17–2.08) (Table 3). After adjusting for all sociodemographic and health covariates, Walk Score^®^ was no longer significantly associated with being overweight or obese, however, neighborhood socioeconomic deprivation remained statistically significant (OR 1.74; 95% CI 1.24–2.43). In this same model, age (age: OR = 1.18; 95% CI 1.10–1.27 + age^2^: OR = 0.99; 0.99–0.99), sex (male: OR = 2.03; 95% CI 1.47–2.81), self-reported mental health (OR = 1.31; 95% CI 1.05–1.62), self-reported physical health (OR = 0.48; 95% CI 0.39–0.60) and attempts to modify weight in the past year (not attempt: OR = 0.37; 95% CI 0.26–0.48) were also associated with being overweight or obese (fully-adjusted model pseudo R^2^ = 0.28).

### 3.5. Correlates of Risk Based on Waist Circumference and BMI Combined

Residing in a socioeconomic disadvantaged neighborhood (OR = 2.16; 95% CI 1.32–3.55), but not Walk Score^®^, was significantly associated with the likelihood of being classified as high risk based on having both a WC of ≥102 cm (men) or ≥88 cm (women) and BMI ≥ 25 kg/m^2^ (Table 3). Adjusting for all covariates, residing in a socioeconomic disadvantaged neighborhood (OR = 2.49; 95% CI 1.42–4.40), age (age: OR = 1.16; 95% CI 1.04, 1.31 + age^2^:)R = 0.00, 95% CI 0.99–0.99), sex (male: OR = 2.05; 95% CI 1.20–3.52), self-reported physical health (OR = 0.42; 95 CI 0.30–0.58) and attempts to modify weight in the past year (no attempt: OR = 0.37; 95% CI 0.22–0.63) were associated with being classified as high risk based on WC-BMI (fully-adjusted model pseudo R^2^ = 0.34) (Table 3).

## 4. Discussion

Contributing to previous evidence [21,22,23], our findings suggest that neighborhood walkability, specifically Walk Score^®^, is negatively associated with waist circumference in Canadian adults. Notably, we found that higher Walk Score^®^ was associated with a lower likelihood of being high risk based on WC, adjusting for neighborhood and individual-level SES, demographic, and health-related characteristics. Our findings support other studies that report negative associations between WC and Walk Score^®^ [25] and other walkability indices [58]. Our results together with previous findings have important public health implications given the elevated health risks associated with having a larger WC [41,42,43].

In several cases, Walk Score^®^ was no longer significantly associated with weight status outcomes after the adjustment for individual characteristics, despite often resulting in only a small attenuation in the estimated log-odds (e.g., BMI-determined overweight; BMI-determined obesity; waist-to-hip ratio high risk, and; WC and BMI combined increased risk). Notably, self-reported BMI is not an indicator of adiposity but rather overall body size and composition (a combination of fat, fat-free muscle mass, other hard and soft tissue, water, height etc.) some of which may be less likely to be influenced by neighborhood characteristics (e.g., height). A reduction in the magnitude of association (odds ratio) towards the null between walkability (an ecological or environment-level correlate) and weight status is expected when adjusting for important individual-level determinants such as sex, age, ethnicity, education, health status, and behavior. Not surprisingly, our findings suggest that neighborhood walkability and SES alone explained less of the variance observed in the weight status outcomes than when individual-level correlates were added to the model. Nevertheless, a one-unit increase in Walk Score^®^ remained significantly associated with decreased odds or about a 1% reduction in likelihood of being high risk based on WC. Waist circumference provides an indirect measure of visceral adiposity and others have found associations between the built environment and visceral adiposity [44]. Walk Score^®^ could be improved by increasing the mix or number of destinations located within a neighborhood or by reducing the distances between home and neighborhood-based destinations. Effective population-level strategies that can prevent weight gain or result in weight loss on a mass scale and reverse the obesity epidemic are desperately needed. Based on US and UK evidence, projection estimates suggest that even a 1% decrease in BMI at the population level could result in significant reductions in the incidence of type 2 diabetes, cancer, and cardiovascular disease [59]. Even small reductions in waist circumference can have significant health benefits [41,60,61]. Thus, urban planning policy that increases neighborhood walkability, even by a small amount, could have a significant population health impact.

Notably, we found that participants residing in a low SES neighborhood were more likely to be identified as high risk based on all weight outcomes independent of Walk Score^®^ and other covariates. Findings from other studies, also suggest that weight status [29,30,31,32] and weight-related behaviors, including physical activity and diet, differ by neighborhood SES. Compared with advantaged SES neighborhoods, disadvantaged neighborhoods are sometimes found to have fewer sidewalks, poorer sidewalk conditions (obstructions and unevenness), and more trash, graffiti, neglected properties [38], fewer recreational facilities [39] and better accessibility to unhealthy food destinations [62] and reduced accessibility to healthy food destinations (e.g., supermarkets) [63]. The finding that neighborhood disadvantage was associated with weight outcomes after controlling for walkability suggests that intermediate factors might be important in the causal pathway. For example, other potential risk factors of overweight and obesity, such as sleep [64] and chronic social stress [65] have been identified, and psychological factors are known to mediate the relationship between SES and health [66]. More research is needed to disentangle the ecological causal pathways by which both the built and socioeconomic characteristics of neighborhoods interact with individual characteristics to determine weight status in adults.

Independent of neighborhood walkability and SES, we also found associations between adverse weight status outcomes and sex, age, self-reported mental and physical activity health, attempts to increase or decrease weight in the past year, and self-reported amount of sleep. The cross-sectional design does not allow us to infer a causal association between weight status and self-reported health and sleep; however, previous cross-sectional and longitudinal studies have found associations between weight outcomes, physical and mental health [67], and sleep [68,69]. Regardless of neighborhood walkability and SES, specific population subgroups (e.g., men, older adults, those with poorer health, and those sleep deficient) may benefit from targeted interventions promoting healthy weight.

The pathways by which Walk Score^®^ is associated with WC cannot be determined from the current cross-sectional study. However, Walk Score^®^ reflects proximity and availability of destinations close to home, which could influence diet and physical activity and subsequently, energy balance and weight. Evidence suggests that proximity to destinations and or having a mix of destinations in the neighborhood such as shops and services (e.g., coffee shops, convenience stores), can support physical activity [70] and impact diet [45] both of which are associated with weight outcomes. For instance, Li et al. [58] found that among older adults, higher neighborhood walkability was associated with lower weight and WC, while higher availability of fast-food restaurants was associated with higher weight and WC. In a subset of our participants who reported their diets, we previously found that the density of food destination was positively associated with diet quality [45]. Furthermore, in Calgary, we have found that neighborhood urban form is indeed associated with physical activity and that higher walkability may encourage higher levels of physical activity [71]. Highly walkable neighborhoods are associated with higher levels of active transportation, which subsequently might increase caloric expenditure and thus promote healthy weight [9]. Policies, programs, and incentives that encourage a mix of destinations (including those offering healthy food options) within walking distance to homes could indirectly decrease overweight and obesity via promoting physical activity and healthy diets. This hypothesis needs to be confirmed using studies that allow temporal causal relations to be assessed (i.e., longitudinal studies and natural experiments).

While we statistically adjusted for important sociodemographic and health characteristics, we were unable to directly adjust for the study population’s preferences or reasons for residing in their current neighborhood that might relate to their physical activity and diet preferences and subsequently impact their weight status (i.e., residential self-selection) [72,73]. Furthermore, the low response rate as well as sample selection bias (i.e., having high levels of education and household incomes) potentially limits the generalizability of our findings. Due to Calgary’s relatively higher incomes, geographical location, climate, history, and population characteristics, the findings of our study may not be generalizable to other Canadian or non-Canadian cities. Despite being validated against other walkability indices and found to be association with physical activity, Walk Score^®^ does not fully capture all built characteristics that may be associated with weight or the behaviors that might determine weight status. Our weight status outcomes might also be impacted on by errors in self-assessment and reporting for WC, HC, and height and weight. Trained researcher or clinician measured WC, HC, and height and weight, and studies including objective measures of adiposity are warranted in the examination of the impact of neighborhood built environment and weight outcomes. While not explored in this study, the mechanisms by which the built environment positively and or negatively impacts weight status directly and indirectly through different behaviors (e.g., physical activity, diet, sleep, sitting) is also critical to the development of effective public and population health interventions and policies [74].

## 5. Conclusions

Our findings suggest that residing in a residential neighborhood with higher walkability is associated with a lower risk of having an unhealthy WC. In other words, neighborhoods that are highly walkable are likely to be less obesogenic. Specifically, a one-unit increase in Walk Score^®^ was associated with a 1% reduction in the likelihood of being at high risk based on WC. Furthermore, our findings suggest that neighborhood-level socioeconomic status should also be considered in developing and implementing policy and interventions for improving weight status.

## Figures and Tables

**Table 1:** Sample profile of sociodemographic, health, neighborhood, and weight status variables (*n* = 851).

Characteristics	Category	Estimate
Age in years (mean (sd))		52.8 (14.3)
Sex (%)	Women	62.4
	Men	37.6
Ethnicity (%)	White	88.4
	Non-white	11.6
Highest education achieved (%)	High school or less	7.6
	College	19.2
	University	73.2
Gross annual household income (%)	<$60,000	10.6
	60–119,000	30.1
	≥120,000	44.1
	Don’t know/refused	15.3
Marital status (%)	Married/common-law	77.3
	Other arrangement	22.7
Number of children <18 years (%)	At least one child	31.0
	No children	69.0
Dog ownership in past year (%)	Owner	32.7
	Non-owner	67.3
Motor vehicle access (%)	Always/sometimes	86.6
	Never/don’t drive	13.4
Smoking in past year (%)	Daily/occasionally	5.3
	Not at all	94.7
Sleep hours/day (mean (sd))		7.3 (1.0)
Self-reported mental health (mean (sd)) ^1^		4.0 (0.9)
Self-reported physical health (mean (sd)) ^1^		3.8 (0.9)
Weight modification in past year (%)	Attempted	51.0
	No attempt	49.0
Waist circumference (WC) (%) ^2^	Low risk	76.1
	High risk	23.9
Waist-to-hip (W-H ratio) (%) ^2^	Low risk	47.8
	High risk	52.2
Body mass index (BMI) (%) ^3^	Healthy weight	59.5
	Overweight	30.2
	Obese	10.3
Combined BMI and WC (%) ^4^	Least risk	56.6
	Increased risk	19.6
	High Risk	14.5
	Very high risk	4.7
	Extremely high risk	4.6
Walk Score (mean (sd))		60.3 (15.0)
Neighborhood SES (%) ^5^	Advantaged	44.5
	Disadvantaged	55.5

^1^ 5-point scale (poor, fair, good, very good, excellent). ^2^ WC: high risk: ≥102 cm for men and ≥88 cm for women; W-H ratio: high risk ≥0.90 cm for men and ≥0.85 cm for women. ^3^ BMI: healthy weight <25, Overweight 25.0–29.9, Obese ≥30. ^4^ BMI/WC: least risk: healthy BMI (<25) and WC <102 cm (men) or <88 cm (women); increased risk: healthy BMI (<25) and WC ≥102 cm (men) or ≥88 cm (women) OR overweight BMI (25.0–29.9) and WC <102 cm (men) or <88 cm (women); high risk: overweight BMI (25–29.9) and WC ≥102 cm (men) or ≥88 cm (women) OR obese BMI (30.0–34.9) and WC <102 cm (men) or <88 cm (women); very high risk: obese BMI (30.0–34.9) and WC ≥102 cm (men) or ≥88 cm (women); extremely high risk: severely obese BMI (≥35). ^5^ Neighborhood SES groups based on grouping the two lowest and two highest quartile categories together.

**Table 2:** Association between (1) waist circumference and (2) waist-to-hip ratio, and neighborhood, sociodemographic, and health variables.

		Waist Circumference High Risk *n* = 233 vs. Low Risk *n* = 618		Waist-to-Hip Ratio High Risk *n* = 444 vs. Low Risk *n* = 407	
Characteristics	Category	Model 1 ^1^ OR (95 CI)	Model 2 ^2^ OR (95% CI)	Model 1 ^1^ OR (95% CI)	Model 2 ^2^ OR (95 CI)
Walk Score^®^		0.98 (0.97–0.99) *	0.99 (0.97–0.99) *	0.99 (0.98–0.99) *	0.99 (0.98–1.01)
Neighborhood SES	Disadvantaged	1.85 (1.32–2.59) *	1.86 (1.26–2.76) *	1.22 (0.92–1.61)	1.39 (0.98–1.97)
	Advantaged	*Ref.*	*Ref.*	*Ref.*	*Ref.*
Age in years			1.25 (1.13–1.37) *		1.10 (1.01–1.19) *
Age in years squared			0.99 (0.99–0.99) *		1.00 (0.99–1.00)
Sex	Men		0.78 (0.53–1.13)		7.92 (5.50–11.41) *
	Women		*Ref.*		*Ref.*
Ethnicity	Non-white		0.66 (0.36–1.21)		1.28 (0.76–2.15)
	White		*Ref.*		*Ref.*
Highest education achieved	High school or less		1.28 (0.67–2.44)		1.44 (0.74–2.80)
	College		1.06 (0.68–1.66)		1.30 (0.84–2.01)
	University		*Ref.*		*Ref.*
Gross annual household income	Don’t know/refused		0.88 (0.50–1.54)		1.25 (0.76–2.07)
	<$60,000		1.21 (0.61–2.39)		1.11 (0.58–2.15)
	60–119,000		1.29 (0.82–2.04)		1.04 (0.69–1.58)
	≥120,000		*Ref.*		*Ref.*
Marital status	Married/common-law		1.06 (0.68–1.66)		1.00 (0.66–1.52)
	Other arrangement		*Ref.*		*Ref.*
Number of children <18 years	No children		1.37 (0.84–2.23)		1.07 (0.71–1.60)
	At least one child		*Ref.*		*Ref.*
Dog ownership in past year	Non-owner		0.90 (0.60–1.34)		1.03 (0.72–1.47)
	Owner		*Ref.*		*Ref.*
Motor vehicle access	Never or don’t drive		1.34 (0.80, 2.26)		1.42 (0.85–2.38)
	Always/sometimes		*Ref.*		*Ref.*
Smoking in past year	Daily/occasionally		0.79 (0.35–1.79)		1.24 (0.60–2.56)
	Not at all		*Ref.*		*Ref.*
Sleep hours/day			0.87 (0.73–1.03)		0.81 (0.69–0.96 )*
Self-reported mental health			1.22 (0.96–1.55)		1.06 (0.85–1.33)
Self-reported physical health			0.45 (0.36–0.57) *		0.74 (0.60–0.92) *
Weight modification in past year	Not attempted		0.37 (0.26–0.54) *		0.61 (0.44–0.84) *
	Attempt		*Ref.*		*Ref.*
	Pseudo R^2^	0.04	0.28	0.01	0.38

*n* = 851. * *p* < 0.05. ^1^ Model 1: Adjusted for Walk Score^®^ and neighborhood SES. ^2^ Model 2: Adjusted for model 1 plus all sociodemographic and health covariates. *Ref*: reference group.

**Table 3:** Associations between (1) body mass index and (2) waist circumference-body mass index combined, and neighborhood, sociodemographic, and health variables.

		BMI Overweight/Obese *n* = 344 vs. Healthy Weight *n* = 507		WC-BMI Increased Risk *n* = 369 vs. Least Risk *n* = 482	
Characteristics	Category	Model 1 ^1^ OR (95 CI)	Model 2 ^2^ OR (95 CI)	Model 1 ^1^ OR (95 CI)	Model 2 ^2^ OR (95 CI)
Walk Score^®^		0.98 (0.97–0.99) *	0.99 (0.98–1.00)	0.99 (0.97–1.00)	0.99 (0.97–1.01)
Neighborhood SES	Disadvantaged	1.58 (1.17–2.08) *	1.74 (1.24–2.43) *	2.16 (1.32–3.55) *	2.49 (1.42–4.40) *
	Advantaged	*Ref.*	*Ref.*	*Ref.*	*Ref.*
Age in years			1.18 (1.10–1.27) *		1.16 (1.04–1.31) *
Age squared			0.99 (0.99–0.99) *		0.99 (0.99–0.99) *
Sex	Men		2.03 (1.47–2.81) *		2.05 (1.20–3.52) *
	Women		*Ref.*		*Ref.*
Ethnicity	Non-white		0.91 (0.55–1.49)		0.51 (0.22–1.22)
	White		*Ref.*		*Ref.*
Highest education achieved	High school or less		0.97 (0.52–1.82)		0.57 (0.21–1.56)
	College		1.28 (0.85–1.93)		0.72 (0.36–1.46)
	University		*Ref.*		*Ref.*
Gross annual household income	Don’t know/refused		1.25 (0.77–2.02)		1.97 (0.91–4.26)
	<$60,000		1.03 (0.55–1.90)		1.34 (0.51–3.54)
	60–119,000		1.18 (0.79–1.76)		1.04 (0.53–2.04)
	≥120,000		*Ref.*		*Ref.*
Marital status	Married/common-law		0.99 (0.67–1.47)		1.03 (0.54–1.96)
	Other arrangement		*Ref.*		*Ref.*
Number of children <18 years	No children		1.46 (0.98–2.17)		2.00 (0.99–4.14)
	At least one child		*Ref.*		*Ref.*
Dog ownership in past year	Non-owner		1.04 (0.74–1.47)		0.68 (0.39–1.20)
	Owner		*Ref.*		*Ref.*
Motor vehicle access	Never/don’t drive		0.84 (0.52–1.36)		1.35 (0.62–2.92)
	Always/sometimes		*Ref.*		*Ref.*
Smoking in past year	Daily/occasionally		0.91 (0.45–1.84)		0.12 (0.01–1.03)
	Not at all		*Ref.*		*Ref.*
Sleep hours/day			0.96 (0.82–1.12)		1.01 (0.79–1.30)
Self-reported mental health			1.31 (1.05–1.62) *		1.33 (0.95–1.87)
Self-reported physical health			0.48 (0.39–0.60) *		0.42 (0.30–0.58) *
Weight modification in past year	Not attempted		0.35 (0.26–0.48) *		0.37 (0.22–0.63) *
	Attempt		*Ref.*		*Ref.*
	Pseudo R^2^	0.03	0.28	0.05	0.34

*n* = 851. * *p* < 0.05. ^1^ Model 1: Adjusted for Walk Score^®^ and neighborhood SES. ^2^ Model 2: Adjusted for model 1 plus all sociodemographic and health covariates. *Ref.*: reference group.

## Declarations

**Ethics approval and consent to participate:** This study complies with the Helsinki Declaration and was granted ethics approval by The University of Calgary Conjoint Health Research Ethics Board. Informed consent was obtained from all study participants; **Consent to publish:** All named authors provided consent to publish this article; **Availability of data and materials:** The datasets generated and/or analysed during the current study are not publicly available due to these data still being used for ongoing research. The survey tools are available on reasonable request.

## Abbreviations

WC	Waist circumference
HC	Hip circumference
BMI	Body mass index
W-H	Waist-to-hip ratio
OR	Odds ratio
CI	Confidence interval
SD	Standard deviation
PAHDQ	Physical activity, health and demographic questionnaire
C-DHQ II	Canadian Diet History Questionnaire II
SES	Socioeconomic status
